# Central blockade of salusin β attenuates hypertension and hypothalamic inflammation in spontaneously hypertensive rats

**DOI:** 10.1038/srep11162

**Published:** 2015-07-29

**Authors:** Hong-Bao Li, Da-Nian Qin, Kang Cheng, Qing Su, Yu-Wang Miao, Jing Guo, Meng Zhang, Guo-Qing Zhu, Yu-Ming Kang

**Affiliations:** 1 Department of Physiology and Pathophysiology, Xi’an Jiaotong University School of Basic Medical Sciences, Xi’an Jiaotong University Cardiovascular Research Center, Xi’an Jiaotong University Health Science Center, Xi’an 710061, China; 2 Department of Physiology, Shantou University Medical College, Shantou 515041, China; 3 Department of Cardiology, Xijing Hospital, Fourth Military Medical University, Xi’an 710032, China; 4 Key Laboratory of Cardiovascular Disease and Molecular Intervention, Department of Physiology, Nanjing Medical University, Nanjing 210029, China

## Abstract

Salusin β is a multifunctional bioactive peptide and is considered as a promising candidate biomarker for predicting atherosclerotic cardiovascular diseases. The present study was designed to investigate the roles and mechanisms of salusin β in the paraventricular nucleus (PVN) in attenuating hypertension and hypothalamic inflammation and whether central salusin β blockade has protective effects in essential hypertension. Normotensive Wistar-Kyoto (WKY) rats and spontaneously hypertensive rats (SHR) were used in this study. The rats were chronic PVN infusion either specific salusin β blocker, antisalusin β IgG (SIgG), or control IgG (CIgG) for 2 weeks. Hypertensive rats had significantly increased salusin β expression compared with normotensive rats. Central blockade of salusin β attenuated hypertension, reduced circulating norepinephrine (NE) levels, and improved cardiac hypertrophy and function in hypertensive rats. Salusin β blockade significantly reduced proinflammatory cytokines (PICs), nuclear factor-kappa B (NF-κB) activity, reactive oxygen species (ROS) levels, and altered renin-angiotensin system (RAS) components in the PVN of hypertensive rats. These findings suggest that the beneficial effects of salusin β blockade in essential hypertension are possibly due to down-regulate of inflammatory molecules and ROS in the PVN.

Hypertension, the major reason of deaths caused by cardiovascular diseases, is an inflammatory state wherein proinflammatory cytokines (PICs), such as tumour necrosis factor-alpha (TNF-α), interleukin (IL)-1β and IL-6, act as neuromodulators and contribute to the hypertensive effect^1^,^2^. In addition to PICs, free radicals, such as superoxide, are also involved in the pathogenesis of hypertension^3^,^4^. Considerable evidence suggests that PICs activate the nuclear factor-kappa B (NF-κB) signaling pathway and increase intracellular reactive oxygen species (ROS), which shifts the intracellular redox status towards NF-κB activation, amplifying the NF-κB signaling pathway^5^. Activation of NF-κB induces gene transcription of PICs, which leads to further increase in ROS production, fostering a positive feedback mechanism, and eventually leading to the progression of hypertension^2^.

Studies over the last several decades have established that the balance between the vasoconstrictor and vasodilatory axis of the renin-angiotensin system (RAS) contributes to the pathogenesis of hypertension^6^,^7^. It is well known that the hypothalamic paraventricular nucleus (PVN) is a key region for the coordination of autonomic and neuroendocrine responses that regulates baroreflex function, salt appetite and sympathetic outflow^8^,^9^. A growing body of evidence suggests that the PVN can synthesize and release both pro- and anti-hypertensive RAS component peptides^6^,^10^. Angiotensin II (Ang II), a principal component of the RAS, induces increased production of PICs and oxidative stress within the PVN, leading to sympathoexcitation and increased blood pressure (BP)^7^. Our laboratory and others have reported that PICs blockade, including TNF-α, IL-1β and IL-6, are capable of regulating various RAS components and attenuating ROS generation in a variety of mammalian tissues, including the heart and the brain^11^,^12^. These studies suggest that cytokines and RAS interact with each other, possibly via production of reactive oxygen species, and thereby regulate BP.

Bioinformatics analyses of a full-length enriched cDNA library originally derived from human cells allowed the prediction that sequences corresponding to an alternative splicing product of the torsion dystonia-related gene (TOR2A) could give rise to a precursor of two potential endogenous bioactive peptides, salusins^13^,^14^,^15^. Salusins are considered to be the source of two bioactive peptides, salusin α and salusin β, which comprise 28 and 20 amino acid residues respectively^13^. Salusin β–like immunoreactivity is localized in vasopressin-expressing neurons of the rat posterior pituitary and hypothalamus^16^. Salusin β accelerates inflammatory responses in vascular endothelial cells^17^ and chronic salusin β infusion into apolipoprotein E-deficient mice enhances atherosclerotic lesions^18^. Intravenous administration of salusin β to rats causes profound hypotension and bradycardia via negative cardiac inotropic and chronotropic actions^19^,^20^. Salusin β in the PVN increases blood pressure and sympathetic outflow via vasopressin in hypertensive rats^13^. Based upon the preceding evidences, salusin β has been shown to be associated with the development and progression of cardiovascular diseases. However, it is not known whether brain salusin β plays an important role in inflammatory response in hypertension, and the underlying molecular mechanisms are far from understanding. In this study, we aim to investigate the role of salusin β in the PVN and to evaluate whether central salusin β blockade has any protective role in cardiac function and hypothalamic inflammation in spontaneously hypertensive rats. We also determined whether the RAS and ROS in the PVN are involved in the effects of salusin β.

## Results

### Effects of antisalusin β IgG

**Figure 1:**
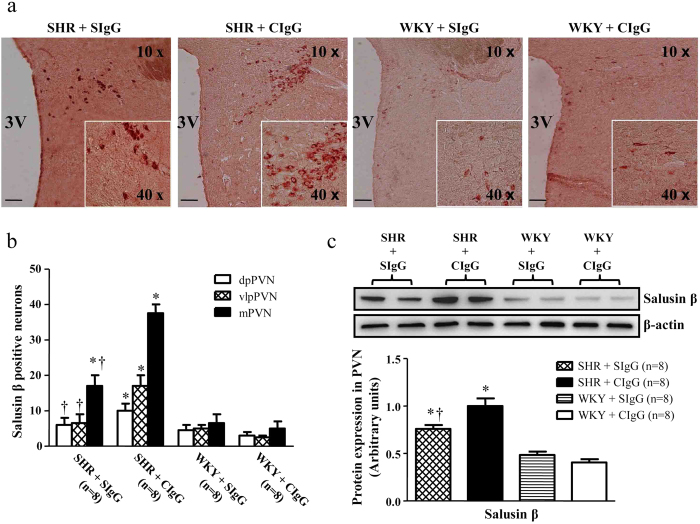
Effects of PVN infusion of control IgG (CIgG) and antisalusin β IgG (SIgG) on the expression of salusin β within the paraventricular nucleus (PVN) in SHR and WKY rats. (**a** and **b**) A representative immunohistochemistry image (×10) and the column diagram showing the effects of PVN infusion of SIgG on the positive neurons of salusin β in the dorsal parvocellular (dpPVN), ventrolateral parvocellular (vlpPVN), and magnocellular (mPVN) subregions of the PVN in SHR and WKY rats. (**c**) A representative immunoblot (top panel) and densitometric analysis (bottom panel) showing protein expression of salusin β within the PVN. (**a**, **b** and **c**) SHR + CIgG rats had significantly higher levels of salusin β within the PVN, whereas SIgG infusion in these rats caused a significant reduction in salusin β expression. Values are mean ± SE. *P < 0.05 versus WKY groups (WKY + SIgG or WKY +CIgG); †*P* < 0.05 SHR + SIgG versus SHR + CIgG. Scale bar: 100 μm. 3 V, third ventricle.

We performed immunohistochemistry staining to determine the corresponding increase in salusin β positive cells expression in the hypothalamic paraventricular nucleus (PVN). As shown in Fig. 1a,b, there was a significant increase in salusin β positive cells expression within the PVN of SHR + CIgG rats when compared with WKY rats. The increase of salusin β protein expression in SHR was further confirmed by western blot analysis (Fig. 1c). Chronic PVN infusion of SIgG caused a significant reduction in salusin β expression in the PVN of hypertensive rats (Fig. 1).

### Salusin β blockade in the PVN attenuates blood pressure in hypertensive rats

**Figure 2:**
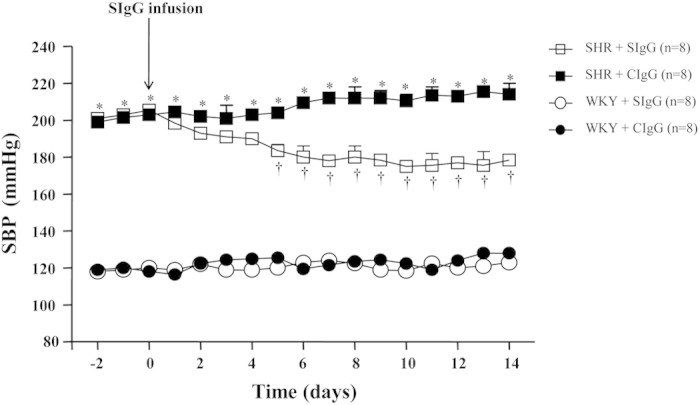
Effects of PVN infusion of SIgG on time course of systolic blood pressure (SBP) in SHR and WKY rats. The SHR + CIgG group had significantly increased SBP when compared with WKY rats. Interestingly, PVN infusion of SIgG in SHR resulted in a significant decrease in SBP, starting from Day 5 of SIgG infusion. Values are mean ± SE. *P < 0.05 versus WKY groups (WKY + SIgG or WKY + CIgG); †*P* < 0.05 SHR + SIgG versus SHR + CIgG.

The systolic blood pressure (SBP) was measured with a noninvasive computerized tail-cuff system (NIBP, ADInstruments, Australia). As shown in Fig. 2, SHR + CIgG rats exhibited a significant increase in SBP when compared with WKY rats (at Day 14, 214 ± 12 vs. 128 ± 2 mmHg, *P* < 0.05). Whereas SHR + SIgG rats exhibited significantly reduced SBP from Day 5, and it remained lower for the duration of the study (at Day 14, 178 ± 2 vs. 214 ± 12 mmHg, *P* < 0.05) when compared with SHR + CIgG rats. CIgG did not have any change in SBP.

The mean arterial pressure (MAP) and heart rate (HR) were measured with a pressure transducer (MLT0380, ADInstruments, Australia) via a catheter in the right carotid artery. The MAP and HR in SHR + CIgG rats were significantly higher than that in WKY rats. There was no significant difference in the body weight between SHR and WKY rats. Chronic PVN infusion of SIgG attenuated MAP and HR in SHR, but not in WKY rats (Table 1).

### Salusin β blockade in the PVN reduces cardiac hypertrophy in hypertensive rats

Echocardiography was used to evaluate left ventricular function and geometry changes. Compared with WKY rats, SHR + CIgG rats had significantly higher interventricular septal thickness (IVSd and IVSs) and left ventricular posterior wall thickness (LVPWd and LVPWs) without modification of left ventricular chamber size (LVEDD and LVESD), fractional shortening and ejection fraction. SHR + CIgG rats had also a higher left ventricles weight to body weight ratio (LVW/BW) compared with WKY rats. Chronic PVN infusion of SIgG significantly reduced interventricular septal thickness and left ventricular posterior wall thickness in SHR when compared with the SHR + CIgG group, indicating reduced cardiac hypertrophy with a central salusin β blockade (Table 2).

### Salusin β blockade decreases PVN inflammation in hypertensive rats

**Figure 3:**
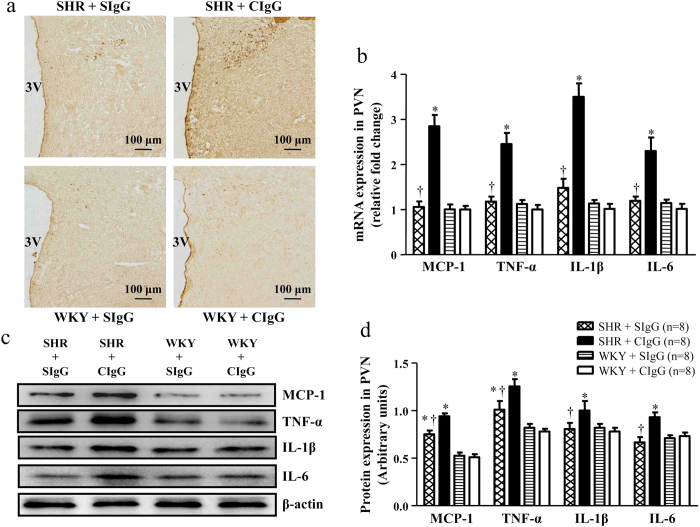
Effects of PVN infusion of SIgG on the levels of PICs within the PVN in SHR and WKY rats. (**a**) Immunohistochemistry for the positive neurons of TNF-α in the PVN in different groups. (**b**) The mRNA expressions of MCP-1, TNF-α, IL-1β and IL-6 in the PVN in SHR and WKY rats. (**c**) A representative immunoblot; and (**d**) densitometric analysis of protein expression of MCP-1, TNF-α, IL-1β and IL-6 in the PVN in SHR and WKY rats. Values are mean ± SE. *P < 0.05 versus WKY groups (WKY + SIgG or WKY + CIgG); †*P*<0.05 SHR + SIgG versus SHR + CIgG. 3 V, third ventricle.

To investigate the effects of chronic blockade of brain salusin β on PVN inflammatory profiles of hypertensive rats, we examined the mRNA and protein levels of PICs (MCP-1, TNF-α, IL-1β and IL-6). We observed that SHR + CIgG rats exhibited marked increases in MCP-1, TNF-α, IL-1β and IL-6 expression in the PVN compared with WKY rats. The up-regulation of MCP-1, TNF-α, IL-1β and IL-6 were significantly attenuated by the SHR + SIgG group. SIgG infusion did not change PICs levels in WKY rats (Fig. 3 and Figure S1a). ELISA studies showed that the levels of TNF-α, IL-1β and IL-6 in the plasma and PVN of SHR were higher than in WKY rats (Table 3), and chronic PVN infusion of SIgG reduced the levels of TNF-α, IL-1β and IL-6 in the plasma and PVN of SHR. There was no significant difference in WKY rats (Table 3).

### Salusin β blockade attenuates oxidative stress in the PVN of hypertensive rats

**Figure 4:**
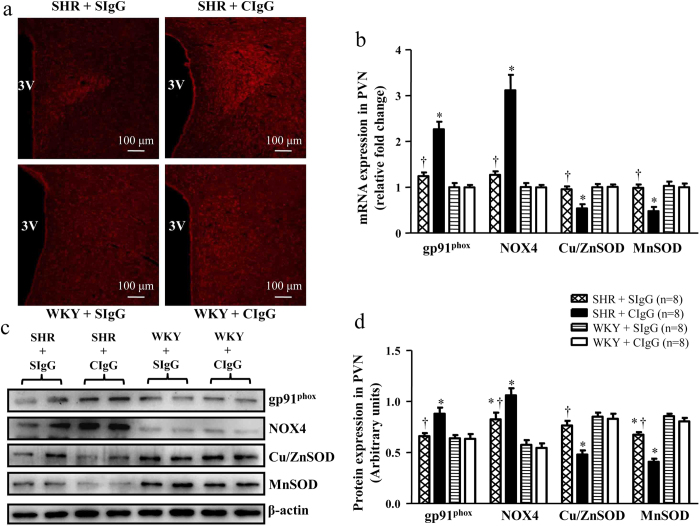
Effects of PVN infusion of SIgG on the levels of oxidative stress within the PVN in SHR and WKY rats. (**a**) A representative immunofluorescence images from the PVN sections of each group showing superoxide production detected by dihydroethidium (DHE) staining (red fluorescence). (**b**) The mRNA expressions of gp91^phox^, NOX4, Cu/ZnSOD and MnSOD in the PVN in SHR and WKY rats. (**c**) A representative immunoblot; and (**d**) densitometric analysis of protein expression of gp91^phox^, NOX4, Cu/ZnSOD and MnSOD in the PVN in SHR and WKY rats. Values are mean ± SE. *P < 0.05 versus WKY groups (WKY + SIgG or WKY + CIgG); †*P* < 0.05 SHR + SIgG versus SHR + CIgG. 3 V, third ventricle.

Immunofl-uorescence revealed that SHR + CIgG rats had more superoxide in the PVN, as determined by fluorescent labeled dihydroethidium (DHE) in comparison with WKY rats (Fig. 4a and Figure S1b). We also observed that SHR + CIgG rats had higher mRNA (Fig. 4b) and protein (Figs. 4c,d) levels of gp91^phox^ and NOX4 (the subunits of NADPH oxidase, major source of induced ROS production), and lower mRNA (Fig. 4b) and protein (Figs. 4c,d) levels of Cu/ZnSOD and MnSOD (the potent superoxide scavenging enzymes, decreased local antioxidant protection is one of the potential sources of ROS formation) when compared with WKY rats. Surprisingly, chronic PVN infusion of SIgG prevented the increase in oxidative stress related markers in the PVN of SHR (Fig. 4).

### Salusin β blockade modulates RAS components in the PVN of hypertensive rats

**Figure 5:**
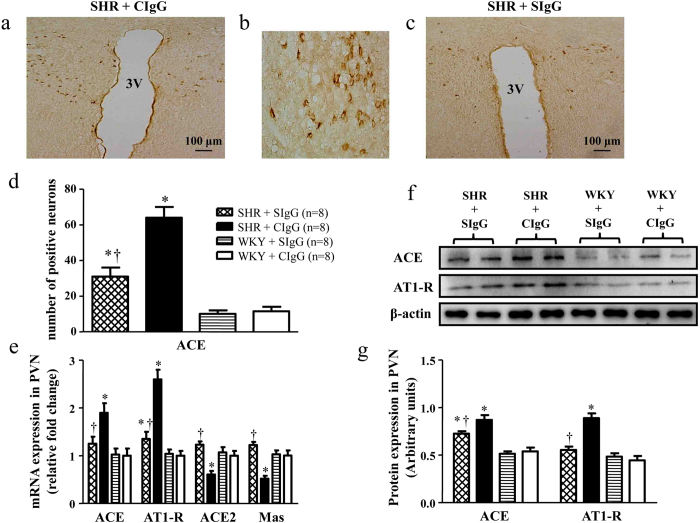
Effects of PVN infusion of SIgG on the level of RAS components within the PVN in SHR and WKY rats. (**a**) Immunohistochemistry for the positive neurons of ACE in a coronal section of the PVN of a SHR. (**b**) High-power view of the section shown in A demonstrating the positive neurons of ACE in the PVN of SHR. (**c**) Effect of salusin β blockade on the ACE expression in the PVN of a SHR. (**d**) Effects of PVN infusion of SIgG on numbers of ACE positive neurons in the PVN of SHR and WKY rats. (**e**) The mRNA expressions of ACE, AT1R, ACE2, and Mas in the PVN of SHR and WKY rats. (**f**) A representative immunoblot; and (**g**) densitometric analysis of protein expression of ACE and AT1R in the PVN of SHR and WKY rats. Values are mean ± SE. *P<0.05 versus WKY groups (WKY + SIgG or WKY + CIgG); †*P* < 0.05 SHR + SIgG versus SHR + CIgG. 3 V, third ventricle.

To determine whether chronic blockade of brain salusin β modulates prohypertensive and antihypertensive components of RAS in the PVN, we examined the levels of ACE, AT1R, ACE2, and Mas receptor in the PVN. We observed that SHR + CIgG rats exhibited higher expressions of ACE and AT1R and lower levels of ACE2 and Mas receptor when compared with WKY + CIgG rats. Chronic PVN infusion of SIgG prevented the increase of ACE and AT1R expressions and up-regulated the expression of ACE2 and Mas receptor in the PVN of SHR (Fig. 5).

### Salusin β blockade attenuates NF-κB activity and p65 levels in the PVN of hypertensive rats

**Figure 6:**
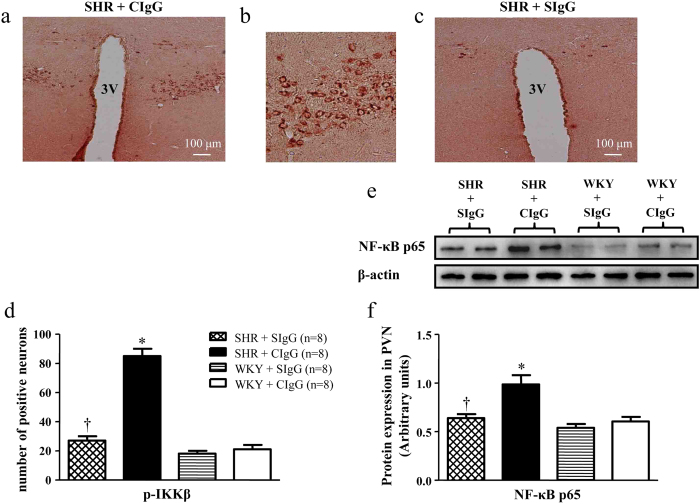
Effects of PVN infusion of SIgG on p-IKKβ positive neurons and NF-κB p65 protein levels in the PVN of SHR and WKY rats. (**a**) Immunohistochemistry for the positive neurons of p-IKKβ in a coronal section of the PVN of a SHR. (**b**) High-power view of the section shown in A demonstrating the positive neurons of p-IKKβ in the PVN of SHR. (**c**) Effect of salusin β blockade on the p-IKKβ expression in the PVN of a SHR. (**d**) Effects of PVN infusion of SIgG on numbers of p-IKKβ positive neurons in the PVN of SHR and WKY rats. (**e**) A representative immunoblot; and (**f**) densitometric analysis of protein expression of NF-κB p65 in the PVN of SHR and WKY rats. Values are mean ± SE. *P < 0.05 versus WKY groups (WKY + SIgG or WKY + CIgG); †*P* < 0.05 SHR + SIgG versus SHR + CIgG. 3 V, third ventricle.

Figure 6a–d showed the positive cells of p-IKKβ (a marker of NF-κB activation) in the PVN in different groups. Figure 6e-f showed protein expression of p65 subunit of NF-κB as assessed by western blot. SHR + CIgG rats had higher NF-κB activity and p65 levels in the PVN than WKY rats. Chronic PVN infusion of SIgG resulted in a significant decrease in NF-κB activity and p65 levels in hypertensive rats, whereas SIgG infusion in WKY rats did not cause any effects on NF-κB activity and p65 expression.

### Salusin β blockade reduced the expression of Fra-like and circulating NE in hypertensive rats

**Figure 7:**
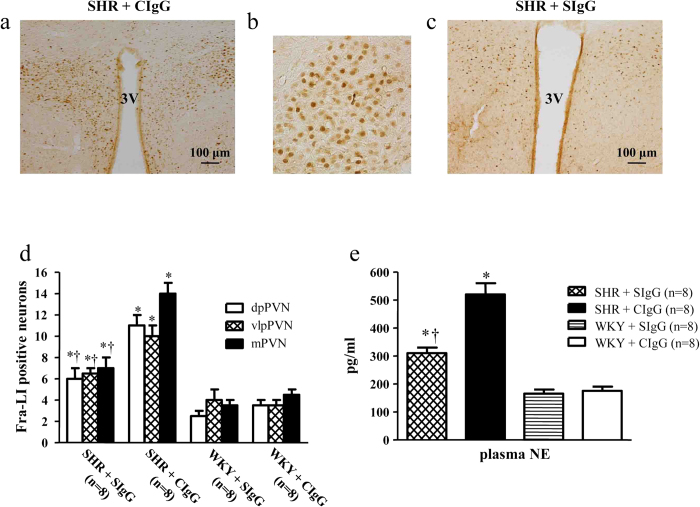
Effects of PVN infusion of SIgG on Fra-like positive neurons within the PVN and plasma NE levels in SHR and WKY rats. (**a**) Immunohistochemistry for the positive neurons of Fra-like in a coronal section of the PVN of a SHR. (**b**) High-power view of the section shown in A demonstrating the positive neurons of Fra-like in the PVN of SHR. (**c**) Effect of salusin β blockade on the Fra-like expression in the PVN of a SHR. (**d**) Effects of PVN infusion of SIgG on numbers of Fra-like positive neurons in the PVN of SHR and WKY rats. (**e**) Plasma NE levels in SHR + CIgG rats were higher than in WKY rats. PVN infusion of SIgG prevented an increased plasma NE levels in SHR. Values are mean ± SE. *P < 0.05 versus WKY groups (WKY + SIgG or WKY + CIgG); †*P* < 0.05 SHR + SIgG versus SHR + CIgG. 3 V, third ventricle.

As shown in Fig. 7, the expression of Fra-like (Fra-LI, fos family gene; indicating chronic neuronal excitation) in the PVN and circulating NE (an indirect indicator of sympathetic activity) significantly upregulated in SHR + CIgG rats compared with WKY rats. Interestingly, SHR + SIgG rats had significantly reduced the expression of Fra-like in the PVN (Fig. 7a–d) and levels of plasma NE (Fig. 7e) when compared with SHR + CIgG rats.

### Co-expression of IL-1β, ACE and gp91^phox^ in the PVN

To check the co-localization of ACE-positive neurons and gp91^phox^-positive neurons in the PVN, we performed double labeling studies using co-focal microscopy. We observed that both ACE and gp91^phox^ were expressed in the neurons of PVN following SIgG infusion (Figure S2). Double labeling results also revealed that 59.8% of the gp91^phox^-positive neurons are also positive for IL-1β in SHR (Figure S3). Only 28.3% of gp91^phox^-positive neurons were positive for IL-1β in the PVN of SHR following with PVN infusion of SIgG (Figure S3).

## Discussion

Salusins, especially salusin β, are expressed and synthesized ubiquitously within human, rat and mouse tissues, including the cardiovascular system and the brain^15^,^19^,^21^. PVN is one of the most important cardiovascular regulatory centers of the brain, which contributes to hypertension development^6^,^22^,^23^.We found that salusin β-like immunopositive neurons and protein level in the PVN were greatly increased in SHR compared with WKY rats. Antisalusin antibody was used to investigate the effects of endogenous salusins because available salusin receptor antagonist has not been found yet^17^,^18^. The specificity of the salusin β staining is assessed by preabsorption of the antibody with the full-length human salusin β, which completely abolishes salusin β staining^14^,^16^. Our present work showed that chronic PVN infusion of antisalusin β IgG (SIgG) reduced the SBP and MAP in SHR, but not in WKY rats. Concomitantly, the significant reductions in circulating plasma levels of NE (an indirect marker of sympathetic activity) and the expression of Fra-LI (indicative of increased neuronal activity) in the PVN in SHR + SIgG rats were observed when compared to SHR + CIgG rats. There are no comparable changes in normotensive rats receiving SIgG. These results indicate that the blockade of endogenous salusin β in the PVN attenuates hypertension and sympathetic activation, which was consistent with the recent finding that microinjection of SIgG in the PVN decreased the renal sympathetic nerve activity (RSNA) and MAP and abolished the effects of salusin β in renovascular hypertension^13^.

Cardiac hypertrophy and dysfunction is considered as an important characteristic of hypertension^24^,^25^. Therefore, we examined whether central salusin β inhibition has cardio-protective effects in hypertensive rats. Our echocardiography data showed that hypertensive rats exhibited cardiac hypertrophy and impaired diastolic function; however, systolic function was not found to be altered as also reported in previous studies^2^. Interestingly, central infusion of SIgG resulted in reduced cardiac hypertrophy in SHR, but not in WKY rats. These results suggest that targeting brain salusin β could be an important therapeutic strategy for cardiac hypertrophy and dysfunction in hypertension.

It is well established that inflammatory molecules play an important role in the pathogenesis of hypertension. Various PICs such as TNF-α, IL-1β and IL-6 have reported to increase with the severity of hypertension^7^,^11^,^22^. Although salusin β plays a vital role in the development of atherosclerotic cardiovascular diseases^15^,^17^,^18^, the changes of salusin β on PVN inflammation of essential hypertension had never been investigated. In our study, the elevated mRNA and protein levels of MCP-1, TNF-α, IL-1β and IL-6 in the PVN of SHR + CIgG rats were observed when compared with normotensive rats. However, chronic SIgG infusion resulted in decreased PVN as well as circulating plasma levels of TNF-α, IL-1β and IL-6 in SHR. The results of this study suggest that salusin β in the brain contributes to PVN inflammation as seen in hypertensive rats.

NF-κB, one of the most important downstream transcription factors responsible for the transcription of PICs, plays an important role in the pathogenesis of cardiovascular diseases, including hypertension^7^,^26^. Additionally, recent studies indicated that salusin β shares the same NF-κB signaling pathways by increased production of PICs in some cardiovascular diseases^17^. Therefore, we speculated that central salusin β inhibition exerts its beneficial effects could be via down-regulation of NF-κB in PVN. In the present study, we observed that chronic PVN infusion of SIgG resulted in down-regulation of NF-κB activity in SHR, but not in WKY rats. Reduced NF-κB activity was also associated with decreased PICs, suggesting that attenuation of NF-κB activity might be attributable to reduced PVN inflammation, which in turn leads to disruption of detrimental positive feed-back cycle involved in cardiac hypertrophy and the progression of hypertension.

It is now well established that an overactivation of the RAS within the brain plays a key role in the pathogenesis of hypertension^6^,^12^. Besides, classical pathway of RAS (Ang II, ACE and AT1R), newly discovered RAS components such as Ang-(1–7), ACE2 and Mas receptor have been shown to play an important role in BP regulation, by counteracting the classical pathway. Recent studies have suggested that the balance between ACE and ACE2, particularly within the brain, is an important factor determining the outcome of hypertension^9^,^22^,^27^. Our results in this study suggested that central blockade of salusin β not only reduced ACE and AT1R levels, but also dramatically upregulated expression levels of ACE2 and Mas receptor within the PVN of hypertensive rats. These results suggest salusin β as an important balance point between the protective (ACE2 and Mas) and nonprotective (ACE and AT1R) arms of the RAS.

Recent investigations have identified that PICs and RAS interact with each other, possibly via induction of reactive oxygen species (ROS), and thereby regulate BP and sympathetic activation^26^,^28^. ROS, in particular superoxide anion (O_2_^•-^), acts as potent intra- and inter-cellular second messengers in signaling pathways causing hypertension^29^,^30^,^31^. Here, central inhibition of salusin β reduced the ROS response within the PVN of hypertensive rats, including O_2_^•-^, thus potentially inhibiting one of the mechanistic pathways by which the hypertensive response and sympathoexcitation (as indicated by decreased plasma NE), is modulated. These results are in accordance with the recent study that salusin β induces the expression of NADPH oxidase-derived ROS in human umbilical vein ECs (HUVECs)^17^.

In summary, our study provides new information that endogenous salusin β in the PVN contributes to hypertension and cardiac hypertrophy in SHR, but not in WKY rats. More importantly, brain salusin β blockade decreases PVN inflammatory molecules and modulates RAS components, possibly through an ROS mediated mechanism, thereby ameliorated the hypertensive response, cardiac hypertrophy and sympathetic activity.

## Methods

### Animals

Ten-week-old male normotensive Wistar-Kyoto (WKY) rats and spontaneously hypertensive rats (SHR) were supplied by Charles River Laboratory Animal Ltd for these experiments. The rats were housed in a climate-controlled room with a 12 h light-dark cycle and allowed access to standard rat chow and tap water *ad libitum*. All animal and experimental procedures in this study were approved by the Animal Care and Use Committees of Xi’an Jiaotong University and were conducted in accordance with the Guide for the Care and Use of Laboratory Animals (National Institutes of Health publication No. 85-23, revised 1996).

### Antisalusin β antibody

The function of salusin β in rats was investigated by rabbit antisalusin β (human) antibody because human salusin β has high homology with the rat salusin β^13^,^14^,^21^. Rabbit antisalusin β (human) serum and rabbit antisalusin β (human) IgG were purchased from Bachem (Bubendorf, Switzerland). The specificity of the rabbit antisalusin β (human) IgG (SIgG) had been determined with radioimmunoassay and no cross-reaction with salusin α. The SIgG is supplied as a lyophilized powder, and is reconstituted by adding 0.01 M PBS (pH 7.4) to get the solution, which was used for chronic PVN infusion. Rabbit anti-salusin β (human) serum was diluted in 0.01 M PBS (1:400) for immunohistochemistry or 5% goat serum (1:1000) for western blot.

### Experimental protocol

Rats were anesthetized with a ketamine (80 mg/kg) and xylazine (10 mg/kg) mixture (ip) and bilateral PVN cannulae were implanted. Following a 7-day surgical recovery, measurement of baseline blood pressure was continuous for 3 days by a tail-cuff occlusion method^6^,^12^. The osmotic minipumps (ALZET, model 1004; infusion rate of 0.11 μL/h) were connected to the PVN cannulae for the continuous infusion of the antisalusin β IgG (SIgG) or control IgG (CIgG) (dissolved in artificial cerebrospinal fluid (aCSF)) directly into the bilateral PVN over 2 weeks. The SIgG dose was determined from previous pilot study in rats where 3 different doses were used (50, 100 and 150 ng/kg/day). The smallest dose (50 ng/kg/day) was found to be no significant effect, whereas the modest dose (100 ng/kg/day) and highest dose (150 ng/kg/day) caused a significant reduction in salusin β expression within the PVN as measured by immunohistochemical staining. We used the lower of the two effective doses of SIgG (100 ng/kg/day) to evaluate the role of salusin β in the PVN in spontaneously hypertensive rats. Controls were infused with control IgG (CIgG, 100 ng/kg/day) through PVN route. The rats were divided into four groups: (n = 25/group): (i) WKY + CIgG; (ii) WKY + SIgG; (iii) SHR + CIgG; and (iv) SHR + SIgG.

### Bilateral PVN cannula implantation for chronic infusion

The rats were implanted with PVN cannula for infusion of SIgG or CIgG, as described previously^6^,^12^,^32^. Briefly, after the rat was anesthetized with a ketamine (80 mg/kg) and xylazine (10 mg/kg) mixture (ip), the head was placed into a stereotaxic apparatus. The skull was then exposed through an incision on the midline of the scalp, and a stainless steel double cannula was implanted into the PVN according to Paxinos and Watson (2007) rat atlas (1.8 mm posterior to bregma, 0.4 mm from midline, and 7.9 mm ventral to dura). The cannula was fixed to the cranium using dental acrylic and two stainless steel screws. A 14 days miniosmotic pump was connected to the infusion cannula through a catheter tube to deliver SIgG or CIgG in the brain and the body of the pump was implanted subcutaneously. Rats received buprenorphine (0.03 mg/kg, sc) immediately following surgery and 12 h postoperatively. The histological identification was made to verify each injection site. The success rate of bilateral PVN cannulation is 68%, and only animals with verifiable bilateral PVN injection sites were used in the final analysis.

### Blood pressure measurements

Blood pressure was determined by a tail-cuff occlusion and acute experiment method. The tail artery systolic blood pressure (SBP) was measured in conscious rats with a noninvasive computerized tail-cuff system (NIBP, ADInstruments, Australia)^13^,^33^.To minimize stress-induced SBP fluctuations, the rats were trained by measuring SBP daily for at least 7 days. To achieve the steady pulse, unanesthetized rats were warmed to an ambient temperature of 32 °C by placing rats in a holding device mounted on a thermostatically controlled warming plate. The SBP values were averaged from ten consecutive cycles per day obtained from each rat.

At the end of the 2th week, rats were anesthetized with a ketamine (80 mg/kg) and xylazine (10 mg/kg) mixture (ip). The femoral artery was cannulated with polyethylene catheters prior filled with 0.1 ml heparinized saline (50 units/ml) and connected to a pressure transducer (MLT0380, ADInstruments, Australia) for continuous mean arterial pressure (MAP) and heart rate (HR) recording. MAP and HR data were collected for 30 min and averaged.

### Echocardiographic assessment of left ventricular function

Echocardiography was performed under ketamine (25 mg/kg, ip) sedation to assess left ventricular (LV) function as previously described^33^. The following parameters were measured: left ventricular end-diastolic diameter and systolic diameter (LVEDD and LVESD, respectively), interventricular septal thickness in diastole and systole (IVSd and IVSs, respectively), left ventricular posterior wall thickness in diastole and systole (LVPWd and LVPWs, respectively). The left ventricular fractional shortening (FS) and ejection fraction (EF) were calculated. All measures were averaged over four consecutive cardiac cycles.

### Collection of blood and tissue samples

At the end of the 2th week of the experiment, rats were anesthetized with a ketamine (80 mg/kg) and xylazine (10 mg/kg) mixture (ip). Trunk blood samples were collected in chilled ethylenediaminetetraacetic acid tubes. Plasma samples were separated and stored at −80 °C until assayed for determination of circulating plasma levels of norepinephrine (NE) and PICs. The brain and heart were harvested, the left ventricles were separated and weighed, and the left ventricles weight (LVW) /body weight (BW) were calculated.

### Tissue microdissection

Microdissection procedure was used to isolate the PVN as previously described^34^,^35^. The tissues were collected from both sides of the PVN of individual rat.

### RNA isolation and real-time RT-PCR

The hypothalamic tissue including PVN was dissected as described previously^36^,^37^. In brief, rat brains were isolated and cut into a coronal segment (−0.92 to −2.13 mm posterior to bregma). From the coronal section we excised a block of the hypothalamus containing the PVN. Total RNA isolation, cDNA synthesis, and RT-PCR were performed as previously described^32^. Total RNA was isolated using RNeasy kits (Qiagen) according to the manufacturer’s instructions, and 1 μg of purified RNA were reverse transcribed with a high-capacity cDNA reverse transcription kit (Bio-Rad). The monocyte chemotactic protein (MCP-1),TNF-α, IL-1β, IL-6, NAD(P)H oxidase subunit 2 and 4 (NOX2 and NOX4), copper/zinc superoxide dismutase (Cu/ZnSOD), manganese superoxide dismutase (MnSOD), angiotensin converting enzyme (ACE), angiotensin II type 1 receptor (AT1-R), angiotensin converting enzyme 2 (ACE2) and Mas receptors (Mas R) mRNA levels were analyzed by quantitative real-time PCR using specific primers (Table 4). The quantitative fold changes in mRNA expression were determined relative to glyceraldehyde 3-phosphate dehydrogenase (GAPDH) mRNA levels in each corresponding group.

### Western blot

The tissue homogenate from the PVN was subjected to Western blot analysis for determination of protein levels of salusin β, MCP-1, TNF-α, IL-1β, IL-6, NOX2 (gp91^phox^), NOX4, Cu/ZnSOD, MnSOD, p65 subunit of NF-κB, ACE, AT1-R, and β-actin. The procedures of Western blot were described previously^12^,^34^. The protein concentration was measured and loaded onto a SDS-PAGE gel and then transferred to a polyvinylidene fluoride membrane. The membrane was then incubated overnight at 4 °C with the primary antibodies. Specific antibodies used included: salusin β, at 1:1000 dilution; MCP-1, TNF-α, IL-1β, IL-6, gp91^phox^, ACE, NOX4, and p65 subunit of NF-κB, at 1:2000 dilution; Cu/ZnSOD and MnSOD, at 1:3000 dilution; and AT1-R, at 1:500 dilution. Antibodies were commercially obtained: salusin β (Bachem, Bubendorf, Switzerland); MCP-1, TNF-α, IL-1β, IL-6, gp91^phox^, ACE, and AT1-R (Santa Cruz Biotechnology, Santa Cruz, CA); Cu/ZnSOD, MnSOD, NOX4, and p65 subunit of NF-κB (Abcam Inc, MA, USA). After washing with wash buffer four times for 10 min each time, blots were then incubated for 1 hour with secondary antibody (at 1:5,000 dilution, Santa Cruz Biotechnology) labeled with horseradish peroxidase. Protein loading was controlled by probing all blots with β-actin antibody (Thermo Scientific, USA) and normalizing their protein intensities to that of β-actin. Band densities were analyzed with NIH ImageJ software.

### Immunohistochemistry and immunofluorescence studies

Immunohistochemistry and immunofluorescence techniques were used to determine the expression of salusin β, TNF-α, IL-1β, gp91^phox^, ACE, phosphorylated IKKβ (p-IKKβ) and Fra-like (Fra-LI, a marker of chronic neuronal activation). The immunostaining protocol used as described previously^9^,^12^,^13^. First brain sections (18 μm) were incubated with 0.3% H_2_O_2_ in methanol for 10 min. Then the sections were incubated with 2% donkey serum in PBS containing 0.3% Triton X100 for 30 min. The sections were incubated with primary antibodie (salusin β, 1:400; TNF-α, p-IKKβ and Fra-LI, 1:50; ACE, IL-1β, and gp91^phox^, 1:100) in 0.01 M PBS at 4 °C overnight. After washing in PBS, sections were further incubated with biotinylated secondary antibodies (at 1:300 dilution, ABC staining system kit, Santa Cruz, CA, USA), Alexa 488-labeled anti-rabbit secondary antibody (at 1:200 dilution, green fluorescence), or Alexa 594-labeled anti-mouse secondary antibody (at 1:200 dilution, red fluorescence) (Invitrogen, CA) for 60 min at room temperature. Immunohistochemistry stained sections were photographed with a conventional light microscopy (DP70, Olympus, Tokyo, Japan). Immunofluorescent staining was visualized with a confocal laser-scanning microscope (Zeiss LSM 710, Carl Zeiss, Inc). For each animal, positive staining cells within the PVN were manually counted in four consecutive sections and an average value was reported. Salusin β-, TNF-α-, or Fra-LI-positive neurons within a window superimposed over the dorsal parvocellular (dpPVN), ventrolateral parvocellular (vlpPVN), and magnocellular (mPVN) subregions of the PVN and were counted similarly for data analysis.

Superoxide generation was determined by fluorescent-labelled dihydroethidium (DHE; Molecular Probes) staining as previously described^7^,^38^. Briefly, dihydroethidium stock (15 mM) was made in DMSO, and dilutions of the stock were used only on the experimental day. Slices containing the PVN were incubated in DHE for 25 min at 4 °C and protected from light. The sections were rinsed three times in PBS and were observed using a conventional light microscopy.

### Detection of plasma norepinephrine (NE)

High-performance liquid chromatography with electrochemical detection (Waters-2465, Waters Corporation, USA) was used for measuring plasma levels of NE as described previously^34^. Briefly, samples or standards were derivatized with o-phtaldialdehyde; 20 μl of the resulting mixture was automatically loaded onto a Novapark C18 reverse-phase column (150 × 4.6 mm, 4 μm particle size, Waters), using a refrigerated autoinjector. The mobile phase consisted of NaH_2_PO_4_ (0.05 M, pH 6.8) with 20% methanol, and the flow rate was 1 ml/min delivered by a Waters pump. The NE concentration was detected and analyzed using Empower 3 analytical software (Waters).

### ELISA studies

Plasma and tissue IL-1β and IL-6 were quantified using commercially available rat ELISA kits (Invitrogen Corporation, CA, USA) according to the manufacturer’s instructions. TNF-α in tissue was measured using a high sensitivity kit (RayBiotech, Inc., GA, USA).

### Statistical analysis

All data are expressed as mean ± SE. The significance of differences between mean values was analyzed by ANOVA followed by a *post hoc* Bonferroni test. BP data were analyzed by repeated measures ANOVA. A probability value of *P* < 0.05 was considered to be statistically significant.

## Additional Information

**How to cite this article**: Li, H.-B. *et al.* Central blockade of salusin β attenuates hypertension and hypothalamic inflammation in spontaneously hypertensive rats. *Sci. Rep.* **5**, 11162; doi: 10.1038/srep11162 (2015).

## Supplementary Material

Supplementary Information

## Figures and Tables

**Table 1:** Changes of body weight, MAP and HR at the end of the 2nd week of the experiment.

**Parameters**	**SHR + SIgG**	**SHR + CIgG**	**WKY + SIgG**	**WKY + CIgG**
N	12	12	12	12
Body Weight, g	326 ± 8	330 ± 5	322 ± 6	325 ± 7
MAP, mmHg	151 ± 7*†	176 ± 8*	106 ± 4	112 ± 6
HR, bpm	354 ± 10†	387 ± 12*	349 ± 9	351 ± 11

Values are means ± SE. MAP, mean arterial pressure; HR, heart rate.

*P < 0.05 vs. WKY groups (WKY + CIgG or WKY + SIgG).

^†^*P* < 0.05 vs. SHR + CIgG.

**Table 2:** Echocardiographic analysis of cardiac hypertrophy and function.

**Parameters**	**SHR + SIgG**	**SHR + CIgG**	**WKY + SIgG**	**WKY + CIgG**
**N**	**12**	**12**	**12**	**12**
LVEDD, mm	6.59 ± 0.14	6.64 ± 0.12	6.58 ± 0.16	6.51 ± 0.13
LVESD, mm	3.44 ± 0.17	3.61 ± 0.24	3.59 ± 0.11	3.62 ± 0.16
IVSd, mm	1.62 ± 0.07†	2.12 ± 0.06*	1.52 ± 0.01	1.49 ± 0.03
IVSs, mm	2.61 ± 0.13†	3.38 ± 0.13*	2.54 ± 0.08	2.59 ± 0.11
LVPWd, mm	1.82 ± 0.05*†	2.21 ± 0.08*	1.61 ± 0.04	1.59 ± 0.03
LVPWs, mm	3.10 ± 0.07†	3.77 ± 0.09*	2.96 ± 0.06	2.99 ± 0.05
FS, %	46.4 ± 2.21	50.1 ± 2.92	42.6 ± 2.31	43.5 ± 2.19
EF, %	79.4 ± 1.36	81.6 ± 1.47	76.9 ± 1.64	78.1 ± 1.55
LVW (g)	0.79 ± 0.06†	1.11 ± 0.05*	0.69 ± 0.05	0.72 ± 0.03
LVW/BW (mg/g)	2.45 ± 0.09†	3.36 ± 0.12*	2.19 ± 0.08	2.21 ± 0.11

Values are means ± SE. LVEDD, left ventricular end-diastolic diameter; LVESD, left ventricular end-systolic diameter; IVSd, interventricular septal thickness in diastole; IVSs, interventricular septal thickness in systole; LVPWd, left ventricular posterior wall thickness in diastole; LVPWs, left ventricular posterior wall thickness in systole; FS, fractional shortening; EF, ejection fraction; LV, left ventricular; BW, body weight.

*P < 0.05 vs. WKY groups (WKY + CIgG or WKY + SIgG).

^†^*P *< 0.05 vs. SHR + CIgG.

**Table 3:** Paraventricular nucleus and plasma levels of proinflammatory cytokines.

**Group**	**PVN (pg/mg protein, n = 10)**			**Plasma (pg/mL, n = 10)**		
	**TNF-α**	**IL-1β**	**IL-6**	**TNF-α**	**IL-1β**	**IL-6**
SHR + SIgG	5.1 ± 0.6*†	31.6 ± 3.7*†	40.2 ± 5.7*†	15.4 ± 2.8†	62.4 ± 7.2†	82.2 ± 9.4*†
SHR + CIgG	7.2 ± 0.9*	48.7 ± 5.1*	60.7 ± 6.9*	30.2 ± 4.1*	112.3 ± 11.8*	121.7 ± 13.5*
WKY + SIgG	2.7 ± 0.2	15.2 ± 2.1	20.3 ± 3.4	11.7 ± 1.9	50.8 ± 6.4	42.3 ± 5.9
WKY + CIgG	2.8 ± 0.3	17.6 ± 2.6	18.5 ± 2.9	12.3 ± 2.2	54.5 ± 6.9	48.3 ± 6.2

Values are means ± SE.

*P < 0.05 vs. WKY groups (WKY + CIgG or WKY + SIgG).

^†^*P* < 0.05 vs. SHR + CIgG.

**Table 4:** Rat primers used for real-time RT-PCR.

**Rat genes**	**Forward (5′-3′)**	**Reverse (5′-3′)**
MCP-1	GTGCTGACCCCAATAAGGAA	TGAGGTGGTTGTGGAAAAGA
TNF-α	ACCACGCTCTTCTGTCTACTG	CTTGGTGGTTTGCTACGAC
IL-1β	GCAATGGTCGGGACATAGTT	AGACCTGACTTGGCAGAGGA
IL-6	TCTCTCCGCAAGAGACTTCCA	ATACTGGTCTGTTGTGGGTGG
gp91^phox^	CTGCCAGTGTGTCGGAATCT	TGTGAATGGCCGTGTGAAGT
NOX4	GGATCACAGAAGGTCCCTAGC	AGAAGTTCAGGGCGTTCACC
Cu/ZnSOD	GGTGGGCCAAAGGATGAAGAG	CCACAAGCCAAACGACTTCC
MnSOD	GGGGATTGATGTGTGGGAGCACG	AGACAGGACGTTATCTTGCTGGGA
ACE	TTGACGTGAGCAACTTCCAG	CAGATCAGGCTCCAGTGACA
AT1-R	CAAAAGGAGATGGGAGGTCA	TGACAAGCAGTTTGGCTTTG
ACE2	ACCCTTCTTACATCAGCCCTACTG	TGTCCAAAACCTACCCCACATAT
Mas	CACTGGCCCTCCTGATGAA	GGATGCCAGAATTGAACACAGA
GAPDH	AGACAGCCGCATCTTCTTGT	CTTGCCGTGGGTAGAGTCAT

MCP-1, chemokine monocyte chemotactic protein-1; TNF-α, tumour necrosis factor-alpha; IL, interleukin; gp91^phox^ (NOX2) and NOX4, NADPH oxidase subunit; Cu/ZnSOD, copper/zinc superoxide dismutase; MnSOD, manganese superoxide dismutase; ACE, angiotensin converting enzyme; AT1-R, angiotensin II type 1 receptor; ACE2, angiotensin converting enzyme 2; Mas, Ang-(1-7) receptor; GAPDH, Glyceraldehyde 3-phosphate dehydrogenase.
