# Supplementary material for: Central blockade of salusin β attenuates hypertension and hypothalamic inflammation in spontaneously hypertensive rats
Source: Sci Rep. 2015 Jul 29;5:11162. doi: 10.1038/srep11162 (PMC4518230; doi:10.1038/srep11162)
Supplement: Supplementary Information [file srep11162-s1.doc]

**Supplementary Data**

**Central blockade of salusin β attenuates hypertension and hypothalamic inflammation in spontaneously hypertensive rats**

Hong-Bao Li^1,a^, Da-Nian Qin^2,a^, Kang Cheng^3,a^, Qing Su^1^, Yu-Wang Miao^1^, Jing Guo^1^, Meng Zhang^1^, Guo-Qing Zhu^4^, Yu-Ming Kang^1^,*

^1^ Department of Physiology and Pathophysiology, Xi'an Jiaotong University School of Basic Medical Sciences, Xi'an Jiaotong University Cardiovascular Research Center, Xi'an Jiaotong University Health Science Center, Xi'an 710061, China

^2^ Department of Physiology, Shantou University Medical College, Shantou 515041, China

^3^ Department of Cardiology, Xijing Hospital, Fourth Military Medical University, Xi'an 710032, China

^4^ Key Laboratory of Cardiovascular Disease and Molecular Intervention, Department of Physiology, Nanjing Medical University, Nanjing 210029, China

Short Title: Role of brain salusin β in hypertension

* Corresponding author: Yu-Ming Kang, M.D., Ph.D.

Department of Physiology & Pathophysiology

Xi’an Jiaotong University Basic Medical Sciences

Xi’an 710061, China

Phone: +86 2982657677

Fax: +86 2982657677

ykang@mail.xjtu.edu.cn


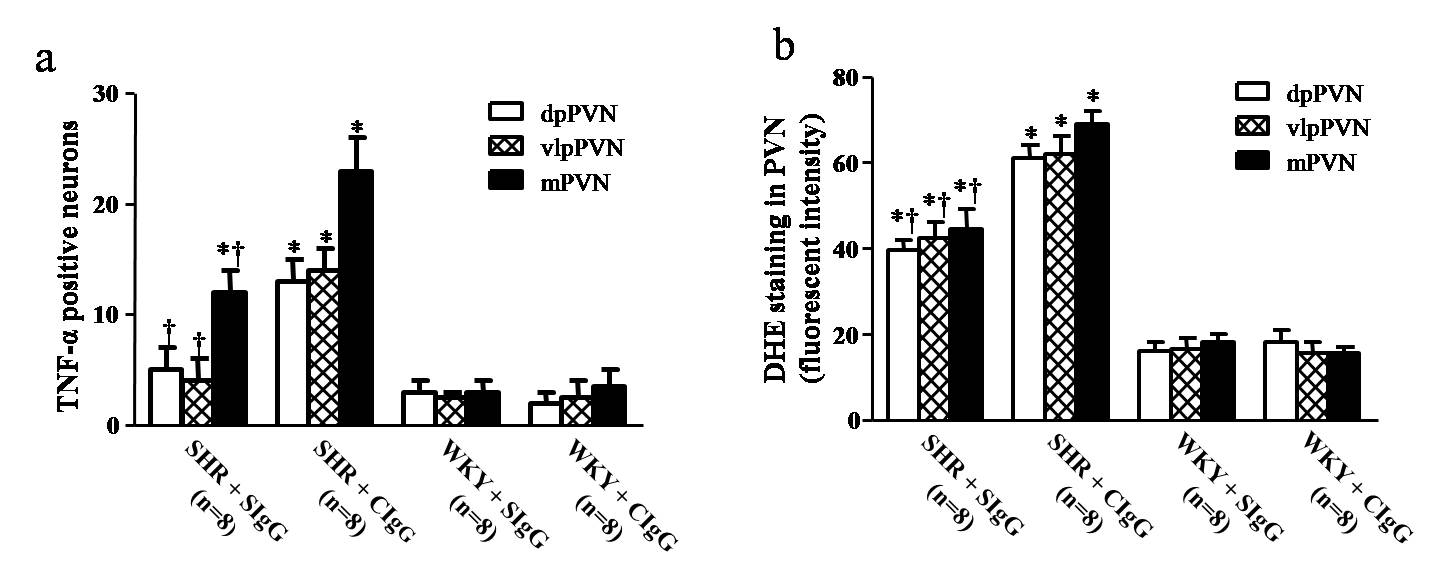


**Figure S1** Effects of PVN infusion of SIgG on the levels of superoxide and the expression of TNF-α positive neurons in the PVN of SHR and WKY rats. (a) Effect of salusin β blockade on numbers of TNF-α positive neurons in the dorsal parvocellular (dpPVN), ventrolateral parvocellular (vlpPVN), and magnocellular (mPVN) subregions of PVN in SHR and WKY rats. (b) Quantitative comparison of DHE fluorescence in the dpPVN, vlpPVN, and mPVN in different groups. Values are mean ± SE. **P*<0.05 versus WKY groups (WKY + SIgG or WKY + CIgG); †*P*<0.05 SHR + SIgG versus SHR + CIgG.


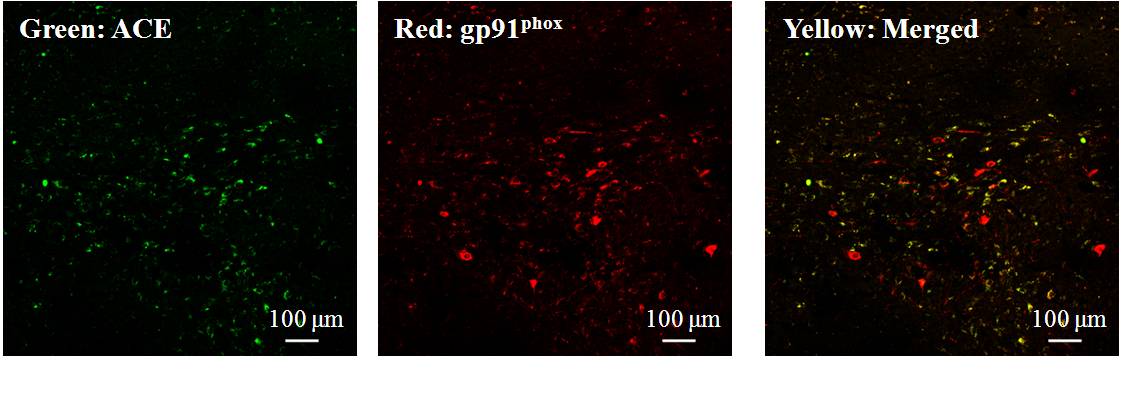


**Figure S2** Laser confocal images showing co-expression of ACE (green) and gp91^phox^ (red) in the PVN of SHR following with PVN infusion of SIgG. The right panel shows the merged images ACE and gp91^phox^.


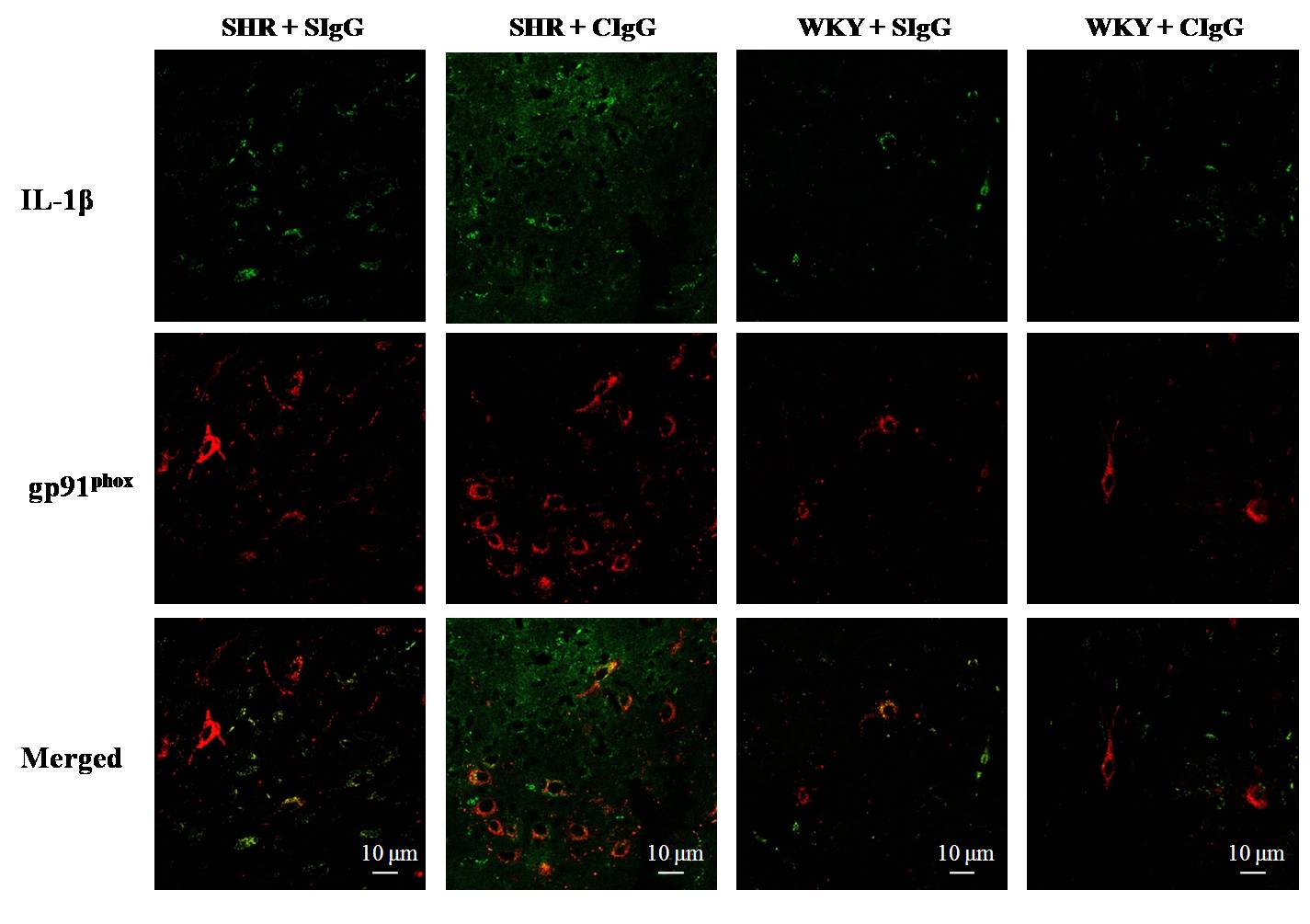


**Figure S3** Effects of PVN infusion of SIgG on the co-expression of IL-1β (green) and gp91^phox^ (red) in the PVN of SHR and WKY rats. The bottom panel shows the merged images (yellow) IL-1β and gp91^phox^.
